# Genetic mechanisms of hemispheric functional connectivity in diabetic retinopathy: a joint neuroimaging and transcriptomic study

**DOI:** 10.3389/fcell.2025.1590627

**Published:** 2025-05-06

**Authors:** Xin Huang, Yu-Xuan He, Song Wan

**Affiliations:** ^1^ Department of Ophthalmology, Jiangxi Provincial People’s Hospital, The First Affiliated Hospital of Nanchang Medical College, Nanchang, Jiangxi, China; ^2^ School of Ophthalmology and Optometry, Jiangxi Medical College, Nanchang University, Nanchang, Jiangxi, China

**Keywords:** DR, resting-state functional MRI (RS-fMRI), AHBA, gene expression, functional homotopy, voxel-mirrored homotopic connectivity (VMHC)

## Abstract

**Background:**

DR represents a major cause of global vision loss; however, the genetic basis of functional homotopy,a critical neurobiological metric reflecting interhemispheric functional synchronization, remains largely unexplored. Emerging evidence suggests that DR patients exhibiting aberrant VMHC may potentially associate with distinct transcriptional profiles. These findings could provide novel mechanistic insights into the neuropathological substrates underlying DR-related visual and cognitive dysfunction.

**Methods:**

Resting-state fMRI data from 46 DR patients and 43 HCs were analyzed to compute VMHC for assessing interhemispheric functional connectivity. Spatial transcriptomic-neuroimaging associations were examined using AHBA, revealing genes significantly correlated with VMHC alterations. Subsequent analyses included functional enrichment assessment and PPI network construction.

**Results:**

DR patients demonstrated significantly lower VMHC in bilateral LING, PoCG, and PreCG versus controls, indicating impaired interhemispheric connectivity in visual-sensorimotor networks. VMHC variations spatially correlated with 4,000 genes (2,000 positive/negative each), enriched in transcriptional regulation, mitochondrial function, synaptic activity (BP/CC/MF), and lipid metabolism/N-glycan biosynthesis (KEGG). PPI network identified hub genes (ACTB/MRPL9/MRPS6,positive; H4C6/NDUFAB1/H3C12,negative) regulating mitochondrial dynamics, cytoskeleton, and epigenetics.

**Conclusion:**

This study represents the first integration of fMRI and transcriptomics to elucidate the genetic determinants underlying VMHC disruption in DR. The findings demonstrate that impaired interhemispheric connectivity in DR involves complex interactions among genes regulating neurovascular, metabolic, and neurodegenerative pathways. These results significantly advance the understanding of neurological manifestations in DR and identify potential therapeutic targets for clinical intervention.

## 1 Introduction

Diabetic retinopathy (DR) is a prevalent microvascular complication among individuals with type 1 or type 2 diabetes, often leading to significant visual impairment (Antonetti et al., 2021). According to estimates from the International Diabetes Federation (IDF), the global population with diabetes is projected to rise from 463 million in 2019 to 700 million by 2045. Meanwhile, the number of adults worldwide affected by DR—the leading cause of blindness among the working-age population—is expected to reach 160 million (Teo et al., 2021). However, most vision loss associated with DR is preventable, and the rates of vision loss related to diabetes and DR have steadily declined over the past few decades (Sabanayagam et al., 2016; Wong et al., 2009). Consequently, as the diabetes population continues to expand, the early prevention and accurate diagnosis and treatment of DR remain significant areas of research. There is a need for more precise evidence to support prevention and treatment guidelines. The retina, an embryological extension of the brain, constitutes a unique window into cerebral health due to its direct neuroanatomical connections with visual cortices and synchronous neurovascular degeneration with central nervous system pathologies (Masland, 2012). A systematic meta-analysis investigating the association between DR and cognitive dysfunction demonstrated a significant correlation between the two conditions. The findings suggest that routine screening for DR could serve as a clinically actionable tool for early identification of individuals at elevated risk of cognitive decline (Cheng et al., 2021). In the latest study, it was also found that there were significant neurodegenerative processes associated with cognitive dysfunction in patients with DR (Oktem et al., 2025). However, the molecular mechanism still needs further study.

Functional magnetic resonance imaging (fMRI) is a non-invasive imaging technique that utilizes the magnetic properties of oxygenated and deoxygenated hemoglobin to detect local changes in blood flow associated with neuronal activity, known as the blood oxygen level dependent (BOLD) response (Ogawa et al., 1992; Zuo et al., 2010). Currently, an increasing number of neuroimaging studies have demonstrated that DR leads to significant functional and structural changes in the brain. Choi et al. reported that the severity of perivascular spaces (PVS) in the basal ganglia (BG) and centrum semiovale, assessed using MRI, may serve as novel imaging biomarkers reflecting the stage of DR and cognitive decline in patients with diabetes (Wong et al., 2009). Huang et al. reported that DR group exhibited reduced efficiency in functional brain networks compared to the healthy control (HC) group (Huang et al., 2019). Qi et al. demonstrated that DR significantly altered neural activity in the middle occipital gyrus, left cerebellum, left inferior temporal gyrus, and left hippocampus (Qi et al., 2020). In addition, Yu et al. found that DR group exhibited different FC patterns between the primary visual cortex (V1) and various vision-related brain regions (Yu et al., 2020). However, the majority of current research focuses on functional and structural changes in specific brain regions.

In this study, we hypothesized that patients with DR may exhibit interhemispheric FC changes in relevant brain regions. The human brain comprises two hemispheres with extensive anatomical and functional connections between homologous sites. Functional homotopy, characterized by a high degree of spontaneous activity synchronization and functional co-activation between geometrically corresponding hemispherical regions, is a fundamental aspect of the brain’s intrinsic functional architecture (Stark et al., 2008). Interhemispheric coupling is closely associated with the processing of motor, auditory, and visual functions. Voxel-mirrored homotopic connectivity (VMHC), an analytical method employed in resting-state functional magnetic resonance imaging (rs-fMRI), relates to the synchronization of spontaneous neural activity between bilaterally symmetric regions in the two hemispheres. In previous neuroimaging studies, Cui et al. reported that interhemispheric FC between the bilateral LING and sensorimotor cortex is reduced in diabetic patients (Cui et al., 2022). In addition, Song et al. found that patients with DR exhibited significant impairment in interhemispheric coordination within the auditory network, visual network, default mode network, and sensorimotor network (Wan et al., 2021). These findings suggest that significant differences in VMHC values between brain regions may reflect distinct neurobiological functions, which could be linked to varying underlying molecular mechanisms.

The Allen Human Brain Atlas (AHBA) utilizes a large tissue microarray analysis of 3,702 distinct samples to provide whole-brain gene expression data for over 20,000 genes (Hawrylycz et al., 2012). Combining whole-brain gene expression data, such as that from the AHBA, with brain imaging data offers a viable approach to identifying the genetic mechanisms underlying various neuroimaging phenotypes (Fornito et al., 2019). This approach also establishes a foundation for bridging the gap between microscopic molecular function and macroscopic brain organization. The method involves correlating spatial gene expression patterns that have been conserved across various brain structures (Hawrylycz et al., 2015). This is despite multiple studies demonstrating the potential of genes as future therapeutic targets for DR and for understanding its pathological mechanisms (Tan and Wong, 2022; Skol et al., 2020; Rowe and Ciulla, 2024).

**FIGURE 1:**
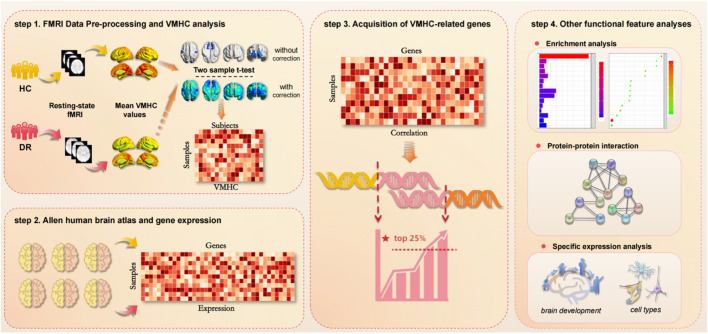
A flowchart of the study design. (step 1) FMRI Data Pre-processing and VMHC analysis. Resting fMRI data from DR and HC were collected to calculate VMHC values. The correlation values are analyzed by Fisher ’z transform, and the zVMHC graph is obtained. Then the two-sample t-test was performed. On this basis, the sample × subject VMHC value matrix is obtained. (step 2) AHBA and gene expression. By using the newly proposed pipeline, gene expression matrices were generated from six donated brains from the AHBA. (step 3) Acquisition of VMHC-related genes. Based on AHBA gene expression data and VMHC differences in samples, transcription-neuroimaging association analysis was performed to identify positive and negative genes associated with VMHC changes in DR patients. (step 4) Other functional feature analyses. Including functional enrichment, specific expression and protein-protein interaction.

This study integrated neuroimaging and gene expression data to investigate the potential neurobiological genetic mechanisms underlying differences in VMHC in the resting brain of patients with DR. Firstly, rs-fMRI data from 46 patients with DR and 43 healthy controls were collected, VMHC values were calculated, and VMHC differences among all groups were compared. The correlation values are analyzed using Fisher’s z transformation, leading to the generation of the zVMHC graph. Subsequently, a two-sample t-test is performed, resulting in a sample × subject VMHC value matrix. The newly proposed pipeline is then utilized to generate gene expression matrices from six donated brains obtained from the AHBA. Transcription-neuroimaging association analyses are carried out based on the AHBA gene expression data and differences in VMHC among the samples to identify both positive and negative genes associated with VMHC changes in patients with DR. Finally, various analyses are conducted to elucidate the potential biological significance of the identified genes, including functional enrichment, specific expression patterns, and protein-protein interactions. The schematic diagram of the research process is presented in Figure 1.

## 2 Materials and methods

### 2.1 Ethics approval

The study protocol adhered to the Declaration of Helsinki and received approval from the Research Ethics Committee of Jiangxi Provincial People’s Hospital. Prior to participating in the study, all participants were provided with detailed information regarding the study’s objectives, methods, and potential risks, and they provided written informed consent for participation.

### 2.2 Participants

In the current study, a total of 89 participants were included, comprising 46 DR patients and 43 HCs, matched in terms of gender, age, and handedness. The participants were recruited from the Department of Ophthalmology at Jiangxi Provincial People’s Hospital. Table 1 diagnostic Criteria and Exclusion Parameters of participants.

**TABLE 1:** Diagnostic Criteria and Exclusion Parameters of participants.

Group	Diagnostic criteria	Examination methods	Exclusion criteria
DR	- Met ADA 2023 diabetes criteria: • FPG≥7.0 mmol/L (126 mg/dL) • RPG≥11.1 mmol/L (200 mg/dL) • HbA1c ≥ 6.5% - DR severity graded per: • ICDRSS(Wilkinson et al., 2003)	- Fundus photography - Fluorescein angiography - SD-OCT	- PDR complications: • Retinal detachment • Vitreous hemorrhage - Ocular comorbidities: • Cataract/glaucoma • High myopia (>-6.0D) • Optic neuritis - Systemic diseases: • Diabetic nephropathy • Cerebrovascular diseases
HC	- Normal glucose metabolism: • FPG 3.9–5.6 mmol/L (70–100 mg/dL) • RPG<7.8 mmol/L (140 mg/dL) • HbA1c<5.7% - No diabetes history	- Dilated fundus exam - SD-OCT - BCVA≥1.0 (Snellen)	- Any ocular pathology - Metabolic disorders - Cerebrovascular disease history

Abbreviations: DR, diabetic retinopathy; HC, healthy control; ADA, American Diabetes Association 2023 Standards; ICDRSS, International Clinical Diabetic Retinopathy Disease Severity ScaleFasting plasma glucose; FPG, fasting plasma glucose; RPG, random plasma glucose; SD-OCT, Spectral-domain optical coherence tomography; BCVA, Best-corrected visual acuity.

### 2.3 MRI acquisition

All participants were instructed to relax, keep their eyes closed, and maintain a motionless state without falling asleep during the rs-fMRI scanning. rs-fMRI data were acquired using a 3T MRI system. Functional images were obtained utilizing a gradient-echo-planar imaging sequence with the following parameters: TR/TE = 2000 ms/25 ms, thickness = 3.0 mm, gap = 1.2 mm, acquisition matrix = 64 × 64, flip angle = 90°, field of view = 240 mm × 240 mm, voxel size = 3.6 mm × 3.6 mm × 3.6 mm, and 35 axial slices.

### 2.4 FMRI data Pre-processing

All rs-fMRI data were preprocessed using DPABI 7.0 (State Key Laboratory of Cognitive Neuroscience and Learning, Beijing, China). The preprocessing included the following steps: (1) conversion of raw DICOM files to NIfTI format; (2) discarding the first 10 functional volumes to account for magnetization equilibration effects and participants’ adaptation to the scanning environment; (3) performing slice timing correction and realignment for head motion correction, with criteria stipulating a maximum displacement of less than 1.5 mm and a maximum rotation of less than 1.5° in the x, y, and z directions; (4) normalizing the images to the Montreal Neurological Institute template (resampling voxel size = 3 mm × 3 mm × 3 mm) using T1 image unified segmentation and smoothing with a 6-mm full-width at half-maximum Gaussian kernel; and (5) applying detrending to remove linear trends. Finally, nuisance covariates, including the Friston 24-parameter model, mean framewise displacement, and average signals from the global brain, cerebrospinal fluid, and white matter, were removed using linear regression.

### 2.5 Voxel-mirrored homotopic connectivity analysis

VMHC is defined as the resting-state FC between any pair of symmetric interhemispheric voxels, specifically the Pearson correlation coefficient between the time series of each voxel and that of its symmetric interhemispheric counterpart. To assess interhemispheric connectivity, VMHC analysis was conducted using the DPABI toolkit. The main steps are as follows: (1) Average all normalized T1 images to obtain mean T1 images; (2) Generate a group-specific symmetric template by averaging the T1 images along with their left and right mirror images; (3) Perform spatial smoothing of the functional data sets using a Gaussian kernel before group analysis; and (4) Convert the obtained VMHC graph into a Z-value graph using Fisher’s Z transformation to improve normality, followed by performing a two-sample t-test analysis.

### 2.6 Brain gene expression data processing

The AHBA dataset provides transcriptome data based on 20,737 genes from six *postmortem* human brains. These genes were derived from 3,702 spatially distinct tissue samples distributed across the cortical, subcortical, brainstem, and cerebellar regions of each brain. The data can be downloaded free of charge through the AHBA Data Portal (http://www.brain-map.org). A white paper available on the AHBA website describes the array design, normalization procedures, and selection of brain samples, as well as anatomical protocols. Compared to other human gene expression maps, AHBA maps samples into stereotactic space, which facilitates the study of subsequent spatial changes. Gene expression data processing begins with updating the probe to the gene annotation. Next, strength-based probe filtering removes probes whose signal does not exceed 50% of the background noise. The probe with the highest expression value for each gene is then selected to represent the gene expression value of that gene. Finally, we obtained the expression values of brain samples, generating a matrix of sample points and genes. Given that the AHBA dataset contained gene expression data solely from two right hemisphere donors and all left hemisphere donor expression data, the VMHC difference map exhibited symmetry between the left and right hemispheres (Hawrylycz et al., 2015). Furthermore, since transcriptional variation across cortical regions is typically less pronounced than that observed between major brain divisions, applying analytical indicators exclusively to cortical samples enables the detection of genes exhibiting more subtle yet consistent expression patterns within functionally defined cortical areas (Hawrylycz et al., 2015). Consequently, in the present study, we restricted our analysis to left hemisphere cortical samples.

### 2.7 Gene expression-VMHC spatial correlation analysis

Initially, the correspondence between gene expression and fMRI data was established. Using the DPABI toolbox, the uncorrected T-value graph was extracted utilizing the MNI coordinates of the AHBA sampling points and the results from the two-sample T-test comparing DR and HC. The average T-value was then calculated to create a matrix graph of the sampling points and their corresponding T-values. Partial least squares regression (PLS) is a regression analysis method suitable for many-to-multilinear regression modeling. It is particularly advantageous when dealing with large numbers of variables in two groups, where multiple correlations exist, and the sample size is small. The model established using partial least squares regression offers benefits that traditional regression analysis methods do not. In this study, we employed the partial least squares regression method to integrate the matrix diagram of sampling points and T-values with the matrix diagram of sampling points and genes to perform a correlation analysis of gene expression and neuroimaging data. Multiple comparisons were corrected by the non-parametric test method (P < 0.05). The number of iterations for spatial autocorrelation correction was set to 5, and the significance threshold for gene selection was established at p < 0.05. Ultimately, we identified the positive and negative gene sets corresponding to the top 13% associated with differences in VMHC.

### 2.8 Gene enrichment analysis

The gene set associated with VMHC differences was analyzed using the DAVID database (DAVID Functional Annotation Bioinformatics Microarray Analysis, ncifcrf.gov). This analysis included functional annotation of Gene Ontology (GO) and enrichment analysis of the Kyoto Encyclopedia of Genes and Genomes (KEGG) pathways. The GO functional annotation encompassed three categories: Gene Ontology Biological Process (GO BP), Gene Ontology Cellular Component (GO CC), and Gene Ontology Molecular Function (GO MF). With a significance threshold set at p < 0.05, the DAVID database analysis results were visualized using the Wei Sheng Xin mapping platform (http://www.bioinformatics.com.cn/). Finally, the Gene Ontology (GO) functional annotation was used to generate bubble charts and bar charts representing the top seven rankings based on P-value significance across the three categories, as well as bar charts depicting the top fifteen rankings based on P-value significance in the KEGG pathway enrichment analysis.

### 2.9 Protein-protein interaction analysis

A protein-protein interaction (PPI) analysis was conducted using the STRING v12.0 functional protein binding network (string-db.org) to evaluate whether VMHC-associated genes can form PPI networks with a minimum interaction score of 0.9 and maximal confidence. In STRING, the range of protein-protein interactions is defined as “functional association,” indicating that two proteins are considered related when there is an evolutionary and specific functional partnership between them (Szklarczyk et al., 2023; Enright and Ouzounis, 2001; Snel et al., 2002; Guala et al., 2020). The top 10% of genes with the highest node degree (i.e., the number of edges connected to a gene) were selected using the CytoNCA plug-in within Cytoscape software and designated as hub genes. Additionally, the spatiotemporal expression patterns of the top three pivotal genes were characterized using the human brain transcriptome database (http://hbatlas.org/).

### 2.10 Gene specific expression

To investigate the genetic loci associated with the affected behavior of patients, we utilized the convergence function available on our website (doughertytools.wustl.edu/CSEAtool.html) to analyze genes related to VMHC differences in terms of tissue specificity. This approach enables a parallel analysis of transcripts enriched in specific cell types, brain regions, and/or developmental windows. Genes exhibiting positive correlations (Figure 7) and negative correlations (Figure 8) were analyzed separately, with specific index thresholds (pSI) set at 0.05, 0.01, 0.001, and 0.0001, respectively.

## 3 Results

### 3.1 Demographics and clinical characteristics

There were significant different in visual acuity between two groups (Table 2).

**TABLE 2:** Demographic and clinical data of participants.

Condition	DR group	HC group	t	p
Gender (male/female)	22/24	22/21	N/A	N/A
BCVA-OD	0.32 ± 0.13	1.05 ± 0.18	−22.629	0.001*
BCVA-OS	0.23 ± 0.16	1.33 ± 0.14	−32.278	0.001*

Note: χ2 test for sex (n). Independent *t*-test for the other normally distributed continuous data (means ± SD). * indicate p < 0.001.

Abbreviations: DR, diabetic retinopathy; HC, healthy control; N/A, not applicable; BCVA, best corrected visual acuity; OD, oculus dexter; OS, oculus sinister.

### 3.2 Comparisons of VMHC between patients with DR and HCs

**FIGURE 2:**
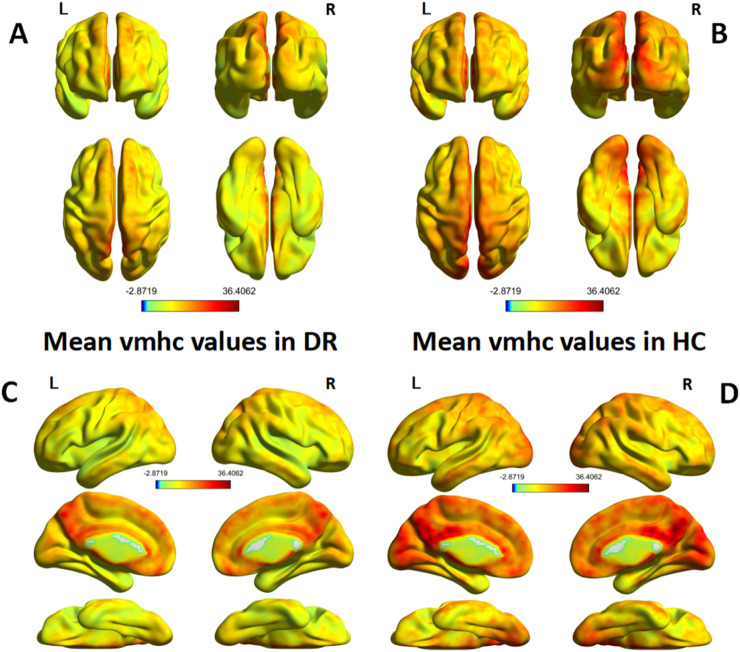
**(A,B)** show the spatial distribution of group mean Z-fraction voxel mirror isotope connectivity (zVMHC) signal values with coronal position in the DR patient group and the HC group, **(C,D)** show the spatial distribution of group mean Z-fraction voxel mirror isotope connectivity (zVMHC) signal values with Sagittal position in the DR patient group and the HC group, respectively. Represents the one-sample t-test results within the two groups, and the color bar represents the t value. HC, Health control; VMHC, voxel image isotope connectivity; L, left; R, right.

**FIGURE 3:**
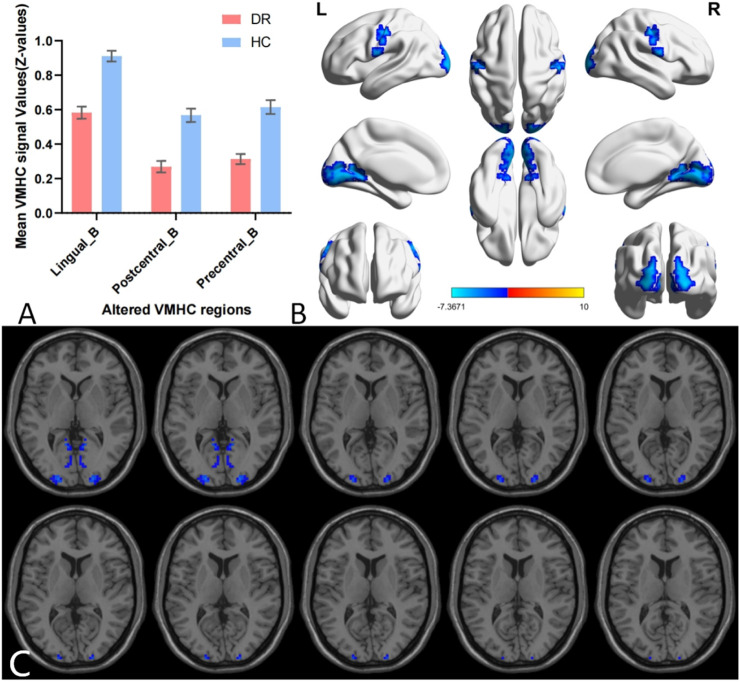
Group comparison of interhemispheric FC between DR patients and HCs. Differences in VMHC signal values between DR patients and HCs. **(A,B)** The mean value of VMHC changes in the DR patient group is shown in histogram format compared to the HC group **(C)**. Blue-indigo color indicates reduced VMHC in DR. Patients with DR displayed significantly reduced VMHC signal values in the bilateral Lingual gyrus (LING), Postcentral gyrus (PoCG) and Precentral gyrus (PreCG) (voxel level p < 0.01, GRF correction, cluster level p < 0.001). GRF, False Discovery Rate; HC, healthy control; L, left; R, right; VMHC, voxel-mirrored homotopic connectivity.

A single-sample t-test was conducted to extract the VMHC results for each group of subjects (p < 0.05). The spatial distribution of group mean Z-fraction voxel mirror isotope connectivity (zVMHC) signal values with coronal position in the DR patient (Figure 2A) group and the HC group (Figure 2B) and with Sagittal position in the DR patient group (Figure 2C) and the HC group (Figure 2D) are illustrated in Figure 2. Details of the brain regions where VMHC values changed between the two groups are provided in Table 3 and Figure 3. Compared to the HC group, the VMHC values of the bilateral LING, PoCG, and PreCG were significantly decreased in DR patients (voxel level p < 0.01, GRF correction; cluster level p < 0.001,GRF,Gaussian Random Field).

**TABLE 3:** Decreased VMHC in patients with DR.

Conditions	Brain regions	MNI			Cluster size	T-score of peak voxel
		x	y	z		
DR < HC	Lingual_B	±6	−66	3	520	−7.3671
DR < HC	Postcentral_B	±63	−6	18	34	−5.5218
DR < HC	Precentral_B	±54	−18	45	74	−5.7778

Statistical value of peak voxels showing significant differences between the two groups. X, Y, Z are the coordinates of primary peak locations in the MNI, space. (voxel level P < 0.01, GRF, corrected for multiple comparisons at a cluster level of P < 0.001). VMHC: voxel-mirrored homotopic connectivity; MNI: montreal neurological institute, B: Both,GRF, Gaussian Random Field.

### 3.3 Genes related to VMHC differences

Spatial correlation analysis was conducted between the matrix of VMHC difference T values for sampling points × groups and the matrix for sampling points × genes. The partial least squares regression method (PLS) was employed, setting the displacement number for spatial autocorrelation correction to 5, with a significance threshold for gene selection set at p < 0.05. Ultimately, we obtained the positive and negative gene sets comprising the top 13% of genes associated with VMHC value differences. Each gene set contained 2,000 genes, which are currently undergoing further analysis.

### 3.4 Genes enrichment results

**FIGURE 4:**
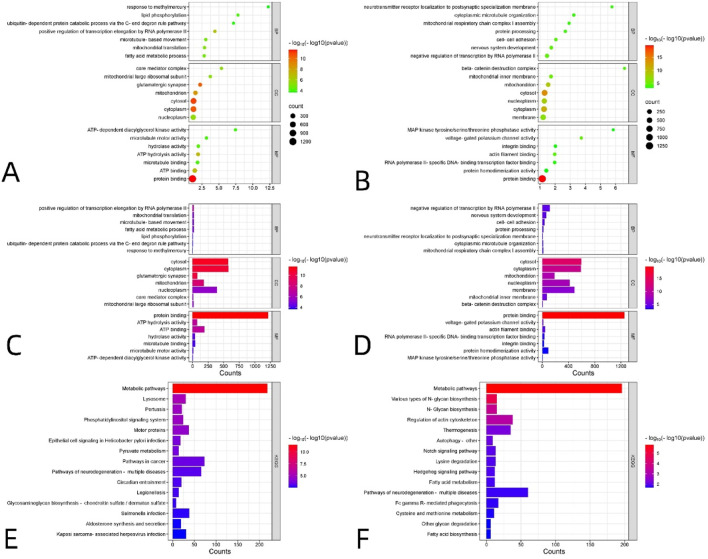
Enrichment analyses of genes reliably associated with VMHC alterations in DR, including 2,000 positively related genes **(A,C,E)** and 2,000 negatively related genes **(B,D,F)**. GO enrichment analysis of related genes. **(A–D)** The y-axis represents GO category and the x-axis denotes GO category score (The ratio of the number of the VMHC-related genes annotated to the item to the number of all genes annotated). The size of each sphere represents the number of genes that overlap with each GO project. The bubble color represents the P-value -log10(P) corrected by FDR-BH, representing the level of significance of enrichment. Enrichment analysis of KEGG pathway of related genes. **(E,F)** MF, molecular function; BP, biological process; CC, cellular component; GO, Gene Ontology; KEGG, Kyoto Encyclopedia of Genes and Genomes. S3 lists details of significant gene enrichment results.

To investigate the role of VMHC difference-related genes in biological processes and the associated biological pathways, we utilized the Wei Sheng Xin mapping platform (http://www.Bioinformatics.com.cn/) for Gene Ontology (GO) and Kyoto Encyclopedia of Genes and Genomes (KEGG) functional enrichment analysis. The results of the functional enrichment analysis are presented in Figure 4.

The results of the positive correlation gene set are presented in Figures 4A,C,E. For the Gene Ontology (GO) analysis shown in Figures 4A,C, the enrichment results for BP primarily include the following: positive regulation of transcription elongation by RNA polymerase II, mitochondrial translation, microtubule-based movement, fatty acid metabolic processes, lipid phosphorylation, ubiquitin-dependent protein catabolism via the C-end degron rule pathway, and response to methylmercury. The enrichment results for CC primarily included the cytosol, cytoplasm, glutamatergic synapse, mitochondrion, nucleoplasm, core mediator complex, and mitochondrial large ribosomal subunit. The enrichment results for MF mainly comprised protein binding, ATP hydrolysis activity, ATP binding, hydrolase activity, microtubule binding, microtubule motor activity, and ATP-dependent diacylglycerol kinase activity. For the KEGG analysis, the enrichment results for biological pathways primarily included metabolic pathways, the phosphatidylinositol signaling system, motor proteins, pyruvate metabolism, and pathways of neurodegeneration across multiple diseases (Figure 4E for details).

The results of the negatively correlated gene sets are presented in Figures 4B,D,F. For the Gene Ontology (GO) analysis shown in Figures 4B,D, the enrichment results for BP primarily include the following: negative regulation of transcription by RNA polymerase II, nervous system development, cell-cell adhesion, protein processing, localization of neurotransmitter receptors to postsynaptic specializations, cytoplasmic microtubule organization, and mitochondrial respiratory chain complex I assembly. The enrichment results for CC primarily included the cytosol, cytoplasm, mitochondrion, nucleoplasm, membrane, mitochondrial inner membrane, and beta-catenin destruction complex. The enrichment results for MF primarily included protein binding, voltage-gated potassium channel activity, actin filament binding, RNA polymerase II-specific DNA-binding transcription factor binding, integrin binding, protein homodimerization activity, and MAP kinase tyrosine/serine/threonine phosphatase activity. For the KEGG analysis, the enrichment results for biological pathways primarily included metabolic pathways, various types of N-glycan biosynthesis, regulation of the actin cytoskeleton, Notch signaling pathway, lysine degradation, Hedgehog signaling pathway, and pathways of neurodegeneration across multiple diseases (Figure 4F for details). S3 lists details of significant gene enrichment results.

### 3.5 PPI network and hub genes

**FIGURE 5:**
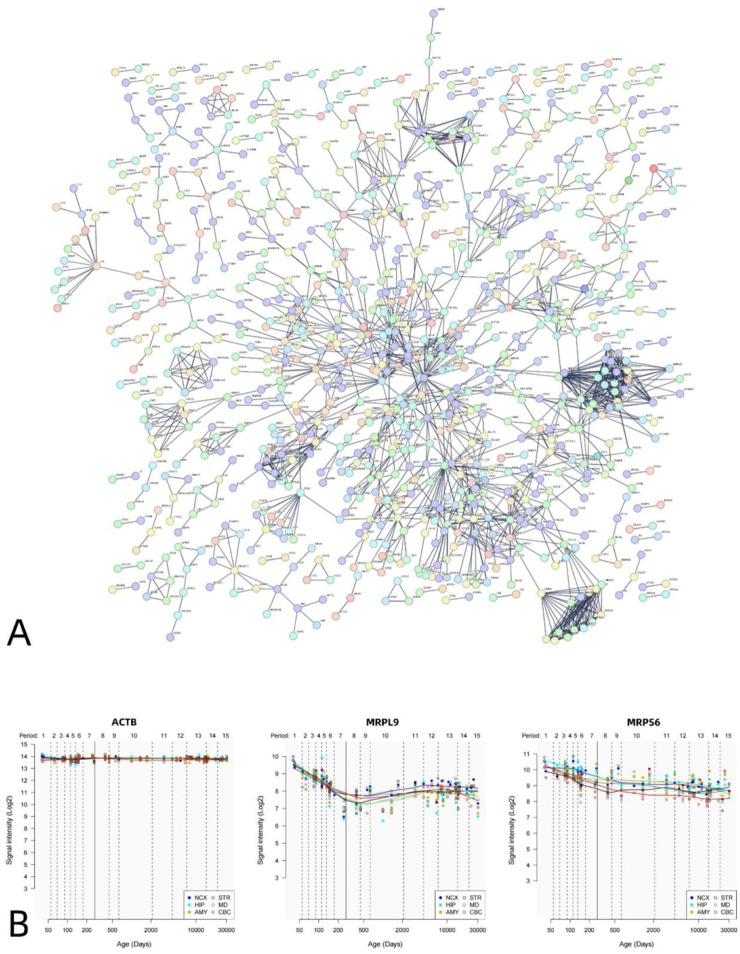
PPI network of positively related genes and hub genes. A statistically significant PPI network was constructed using positive genes related to VMHC. The PPI network consists of 1830 nodes. Each node represents a protein. The minimum required interaction score is a maximum confidence of 0.900 **(A)**. Spatial–temporal specific expression curves of three hub genes with the highest degree values (ACTB, MRPL9, MRPS9) **(B)**.

**FIGURE 6:**
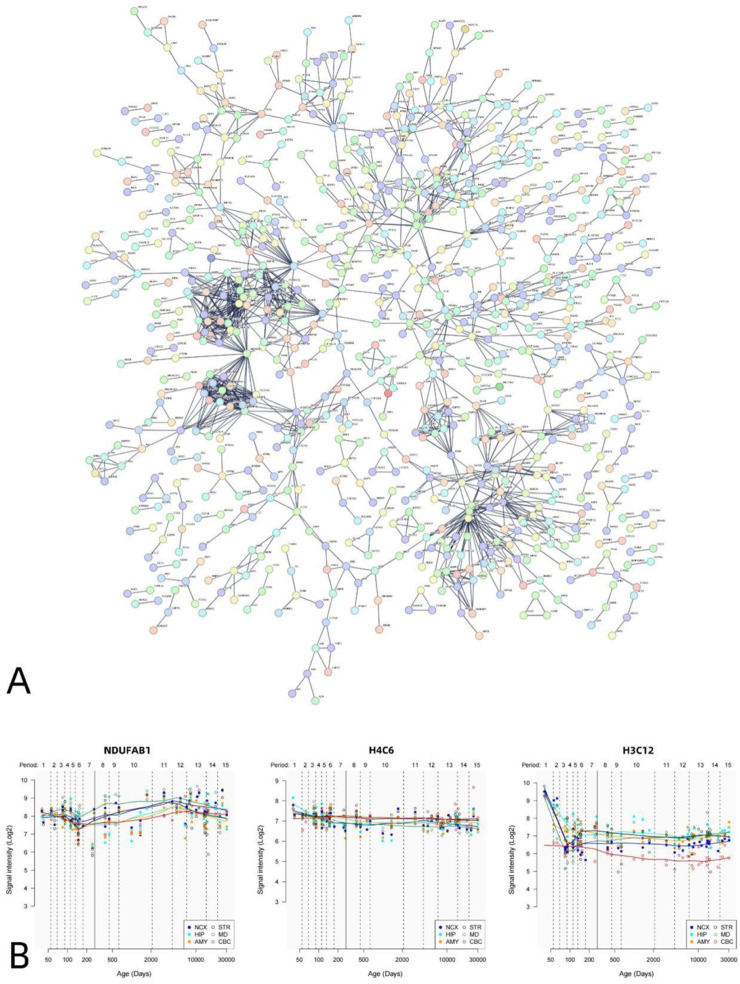
PPI network of negatively related genes and hub genes. A statistically significant PPI network was constructed using negative genes related to VMHC. The PPI network consists of 1817 nodes. Each node represents a protein. The minimum required interaction score is a maximum confidence of 0.900 **(A)**. Spatial–temporal specific expression curves of three hub genes with the highest degree values (NDUFAB1, H4C6, H3C12) **(B)**.

The PPI analysis revealed that the differential genes associated with VMHC could construct PPI networks. The set of positively correlated genes is illustrated in Figure 5, while the PPI network diagram (Figure 5A) comprises 1,812 nodes (PPI enrichment p-value: < 6.44e-12). Figure 6 presents the negative correlation gene set, while the PPI network diagram (Figure 6A) consists of 1,817 nodes, indicating a PPI enrichment p-value of less than 1.0e-16. Both results were statistically significant. The top 10% of genes identified by CytoNCA in Cytoscape software, based on the highest node degree, are designated as hub genes. Additionally, we delineate the spatial and temporal expression profiles of the three most representative hub genes. The genes exhibiting positive correlation are ACTB, MRPL9, and MRPS6 (Figure 5B), while the genes demonstrating negative correlation are H4C6, NDUFAB1, and H3C12 (Figure 6B).

### 3.6 Gene specific expression results

**FIGURE 7:**
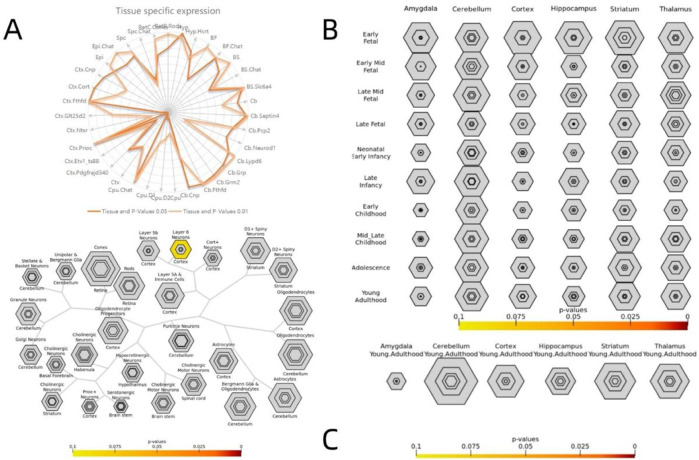
Specific expression analysis of the positive genes related to VMHC. Varying stringencies for enrichment (pSI) are represented by the size of the hexagons going from least specific lists (outer hexagons) to most specific (center). The color bars represent P-values Specific expression analysis across cell types **(A)** Specific expression analysis across adult brain regions. **(B)** Specific expression analysis across brain regions and development. **(C)** S1 lists Fisher exact P-values for specific analysis results of positive correlation genes.

**FIGURE 8:**
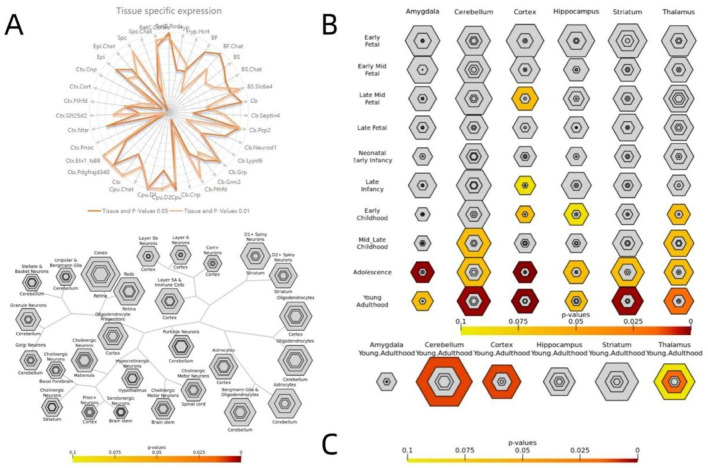
Specific expression analysis of the negative genes related to VMHC. Varying stringencies for enrichment (pSI) are represented by the size of the hexagons going from least specific lists (outer hexagons) to most specific (center). The color bars represent P-values Specific expression analysis across cell types **(A)** Specific expression analysis across adult brain regions. **(B)** Specific expression analysis across brain regions and development. **(C)** S2 lists Fisher exact P-values for specific analysis results of negative correlation genes.

The specific expression results of 2,000 positive and negative genes associated with VMHC changes in DR patients are presented in Supplementary Material 1. Cross-cell type expression analysis revealed that positively correlated genes were specifically expressed in cortical neurons (Figure 7A). Region-specific analysis of the adult brain indicated that negatively correlated genes were specifically expressed in the cerebellum, cortex, and thalamus (Figure 8C). Specific expression analysis across brain regions and developmental stages revealed that negatively associated genes are preferentially expressed during adolescence and young adulthood (Figure 8B). Fisher’s exact p-values for the specific analysis results of the two related gene sets are provided in Supplementary Files S1 (including the results from Figure 7) and Supplementary Files S2 (including the results from Figure 8).

Neurobiological genetic signature that underlies brain abnormalities in individuals with DR.

## 4 Discussion

To our knowledge, this is the first study to employ a comprehensive analysis of brain imaging and gene expression data to investigate the genetic mechanisms underlying VMHC changes in DR patients. By analyzing rs-fMRI signals, we found that VMHC values in the bilateral LING, PoCG, and PreCG were significantly lower in DR patients than in healthy controls. Additionally, transcriptome-neuroimaging correlation analysis demonstrated that the variation in VMHC values was spatially correlated, both positively and negatively, with multiple genes. Among these, 2,000 genes (approximately the top 13% of the relevant gene set) were selected for follow-up analysis. These genes were enriched in significant BP, CC, and MF, as well as in the Kyoto Encyclopedia of Genes and Genomes (KEGG) biological pathways within the cerebral cortex. Furthermore, the PPI analysis revealed that differential genes associated with the ventricular myocardium heart condition (VMHC) could be utilized to construct PPI networks. The results from both the positively and negatively correlated gene sets demonstrated statistical significance (PPI enrichment p < 6.44e-12). Additionally, these genes exhibit specific expression patterns across various cell types and developmental stages in the brain. The gene set initially identified in this study encompasses diverse functional categories, including transcriptional regulation, oxidative stress response, and metabolic pathways. These findings align with existing evidence suggesting that DR represents a systemic metabolic disorder involving multiple brain regions. Although the genes associated with VMHC have indirect connections with neurotransmitters, synaptic functions, and neurodegenerative pathways, the core KEGG pathways (such as glycolysis, mitochondrial function, and lipid metabolism) and molecular functions (such as oxidative stress response and energy metabolism regulation) in which these genes are enriched indicate that they are primarily involved in metabolic regulation. This is highly consistent with the pathological mechanism of DR as a systemic metabolic disorder. The differential gene expression in cognitive - related brain regions may be a manifestation of secondary neural damage caused by metabolic abnormalities. In the present analysis, we focused on elucidating significantly enriched pathways and identifying core regulatory genes associated with DR pathogenesis. This provides a foundational basis for investigating the underlying neurobiological genetic mechanisms associated with VMHC variations in individuals with DR.

Initially, we identified that the DR group exhibited reduced VMHC values in the LING between the brain hemispheres. Situated in the primary visual cortex, the LING is a critical component of the dorsal visual pathway, facilitating not only visuospatial processing but also object and face recognition, as well as memory formation (Rehman and Al Khalili, 2024). The most prominent characteristic of DR patients is the presence of varying degrees of visual impairment, which can lead to blindness in severe cases. In previous studies on DR, Qi et al. found that patients with DR had significantly reduced FC within the visual cortex compared to HC group (Rehman and Al Khalili, 2024). In other ocular disorders, abnormal decreases in VMHC values have been observed in the same visual cortex. Liu et al. reported in a study on strabismus and amblyopia that the functional connectivity values of the bilateral BA17 region and the left cuneus (bilateral LING) were reduced (Liu et al., 2022). Additionally, Shu et al. reported that the functional connectivity of the tongue and wedge network is significantly reduced in patients with primary angle-closure glaucoma (PACG) (Shu et al., 2023). Consequently, we hypothesize that dysfunction of the visual cortex in patients with ocular diseases may result in visual impairments. The reduced VMHC values in the LING among DR patients indicate diminished functional connectivity between hemispheres, suggesting that information processing and coding in the bilateral primary visual cortex may be compromised. Furthermore, our study revealed reduced VMHC values in the central gyrus before and after the onset of DR. The anterior central gyrus (BA4) and posterior central gyrus comprise the principal motor cortex (M1) and principal sensory cortex (S1), respectively, serving as key regions within the somatomotor network (SMN). SMN plays a critical role in the regulation of eye movement (Iacoboni et al., 1997) and is involved in encoding the activity of the oculomotor nerve (Blanke et al., 2000). Prior research has established a significant association between the SMN and spontaneous brain activity in the primary visual cortex (Wang et al., 2008). This strong correlation between the two components plays a pivotal role in the processing of spatial visual information (Ringach et al., 1996). Hence, we postulate that subsequent to visual impairment, the SMN is impacted by decreased visual input, suggesting potential abnormalities in the spatial visual information processing of patients with DR. The reduced VMHC values in LING, PoCG, and PreCG may serve as non-invasive neuroimaging biomarkers for early detection of DR-related neural dysfunction, particularly in patients with subclinical visual impairment. This could complement existing ophthalmological assessments (e.g., fundus photography) to improve diagnostic accuracy.

The diabetic environment disrupts metabolic homeostasis and also alters various genes, including those associated with oxidative stress, apoptosis, and inflammation. Consistent with our findings, in gene enrichment analysis, we found that VMHC difference-related genes were mainly enriched in gene transcriptional regulation, mitochondrial structure and function, neurotransmitters, synapses and nervous system development, metabolic pathways, and various neurodegenerative disease pathways. Gene transcription is a fundamental biological process wherein an organism’s genetic code is interpreted as a series of instructions governing cell division, differentiation, migration, and maturation. In their study on diabetic kidney (DN) and DR, Xu et al. showcased that elevated chemokines could potentially serve as biomarkers for the early detection of DR through the analysis of integrated single-cell transcriptomics (Xu et al., 2023). Chemokines are widely recognized for their crucial involvement in immune system development and homeostasis, influencing both protective and detrimental immune and inflammatory responses (Hughes and Nibbs, 2018). In alignment with our findings, the KEGG enrichment analysis of upregulated genes revealed significant enrichments in pathways associated with bacterial and viral infections. Mitochondria play a pivotal role in regulating synaptic and neural plasticity, crucial for the formation of new neuronal synapses and pathways (Knott et al., 2008). Moreover, they play a role in regulating cellular calcium homeostasis (Rizzuto et al., 2012), managing oxidative stress (Bhatti et al., 2017), and controlling apoptosis (Abate et al., 2020). Evidence supports the pivotal role of mitochondrial dysfunction and oxidative stress in the pathogenesis of DR (Xu et al., 2023). Oxidative stress can damage the integrity of cell membranes, induce apoptosis, microvascular damage and barrier damage (Bonnefont-Rousselot, 2002). Concurrently, the rise in oxidative stress during hyperglycemia can impair the structure and function of mitochondria, leading to the generation of mitochondrial ROS (Santos et al., 2012), endothelial cells and pericytes apoptosis (Kowluru, 2013), and ultimately lead to the occurrence of DR. In the nervous system, neuronal communication primarily takes place at synapses, facilitating rapid transmission of information between neurons (Pereda, 2014). Analysis of gene enrichment revealed significant associations with processes such as microtubule-based movement, actin filament binding, and the regulation of actin cytoskeleton. Notably, our results highlighted a significant enrichment of genes related to glutathione synapses. Glutathione, a crucial neurotransmitter in the antioxidant system, has been shown to be downregulated in the pathogenesis of DR (Kowluru et al., 1997). Moreover, selenium-dependent glutathione peroxidase, particularly GPx1 and GPx4, are predominantly found in mitochondria. The depletion of GPX4-GSH is strongly linked to ferroptosis, an iron-dependent form of cell death frequently observed in DR (Liang et al., 2009; He et al., 2023). In the KEGG analysis, gene enrichment was linked to various pathways related to neurodegeneration. Retinal neurodegeneration is characterized by reactive gliosis, neuronal dysfunction, and neuronal loss (Bianco et al., 2022). Multiple studies have demonstrated that in the early stages of DR, the retinal neurovascular coupling mechanism is impaired (Bek, 2017). In a previous study by M. S. Roy et al., it was demonstrated that patients with DR had photoreceptor dysfunction (Roy et al., 1986). Furthermore, Takahashi et al. assessed the inherent condition of retinal nerve fiber layer damage in diabetes (Takahashi et al., 2006). Our study’s identification of genetic determinants underlying VMHC alterations in DR not only reveals novel therapeutic targets but also underscores the imperative of incorporating neuroprotective strategies into comprehensive DR management protocols.

Enrichment analysis reveals that disruptions in various metabolic pathways offer valuable insights into the genetic mechanisms underlying DR pathogenesis. Preretinal neovascularization is the leading cause of severe vision impairment in individuals with DR, characterized by the accumulation of advanced glycosylation end products. Initial studies have indicated that N-linked glycosylation plays a crucial role in angiogenesis, as inhibiting enzymes early in the glycosylation pathway can impede blood vessel formation (Tiganis et al., 1992). Angiogenesis is primarily mediated by vascular endothelial growth factor (VEGF) receptors (Otrock et al., 2007) and integrins (Silva et al., 2008). Like many cell surface proteins, VEGF and integrin receptors undergo glycosylation. Anna I. Markowska et al. discovered that Gal-3 regulates neovascularization by binding to N-sugars produced by integrin αvβ3, thereby activating signaling pathways that facilitate the formation of new blood vessels (Markowska et al., 2014). Our hypothesis suggests that N-Glycan biosynthesis plays a significant role in elucidating the molecular genetic mechanism of visual impairment in patients with DR. New molecules specifically targeted to inhibit glycosylation metabolism may open up new possibilities for treating DR (Bousseau et al., 2018). Hyperglycemia is a prominent characteristic of diabetes, leading to abnormal pyruvate metabolism, which can be indicative of glucose metabolism dysfunction and mitochondrial impairment. Rajala et al. observed alterations in both metabolic and non-metabolic functions in mice within the DR Group. Metabolic functions showed elevated pyruvate kinase activity, higher lactate levels, and decreased oxidative redox rates, leading to diminished mitochondrial oxidation. Non-metabolic functions demonstrated a decline in the expression of the pyruvate kinase M2 subtype (PKM2), contributing to weakened rod function and visual impairment in diabetic patients (Rajala et al., 2020). Therefore therapeutic modulation of pyruvate kinase M2 (PKM2) expression demonstrates considerable clinical potential for attenuating visual deterioration in DR patients (Weh et al., 2023). Furthermore, longitudinal assessment of PKM2 activity may serve as a valuable biomarker for monitoring disease progression and predicting visual acuity outcomes in DR. Besides hyperglycemia and hypertension, recent studies have indicated that disturbances in lipid metabolism may also play a significant role as pathogenic factors in the onset and progression of DR (Eid et al., 2019). Polyunsaturated fatty acids, particularly arachidonic acid (AA) and its derivatives (Iyer et al., 2021), serve as crucial mediators in the pathogenesis of Proliferative Diabetic Retinopathy (PDR), playing vital roles in inflammation and angiogenesis processes (Schwartzman et al., 2010). Furthermore, studies have indicated that diabetes-induced reductions in docosahexaenoic acid (DHA) and elevations in pro-inflammatory omega-6 polyunsaturated fatty acids play a role in fostering the progression of DR through diverse mechanisms (Connor et al., 2007; Tikhonenko et al., 2013; Tikhonenko et al., 2010). Consequently, beyond conventional pharmacological and surgical interventions for DR patients, implementing personalized nutritional strategies guided by lipidomic profiling may play a pivotal role in comprehensive disease management. In summary, our gene enrichment analysis has identified fundamental molecular biomarkers associated with DR pathogenesis. Importantly, the elucidated metabolic pathway dysregulations provide novel mechanistic insights that may inform the development of targeted therapeutic and preventive strategies for DR management.

PPI analysis revealed that genes linked to VMHC in DR patients formed a PPI network, whereby hub genes were identified based on their node degree being within the top 10%. These hub genes are pivotal in various critical biological processes, highlighting their significance. Investigating the association between key genes and diseases proves beneficial for delving into the underlying genetic mechanisms of DR. DR is a metabolic disorder characterized by hyperglycemia. In a study focused on genes associated with diabetic glomerular sclerosis, actin beta (ACTB) emerged as one of the most reliable reference genes in rat mesangial cells exposed to high glucose conditions (Biederman et al., 2004). ACTB plays a critical role in cell structure, movement, and integrity, owing to its essential involvement in various cellular functions. Therefore, this gene is abundantly expressed in numerous cell lines (Bugyi and Kellermayer, 2020). A genetic analysis focused on retinal development identified beta-actin as crucial for the maintenance of proper retinal structure and function (Rocha-Martins et al., 2012). MRP, a nuclear gene, is initially synthesized in the cytoplasm before being translocated to the mitochondria for assembly. Increasing evidence supports its role not only in mitochondrial oxidative phosphorylation (OXPHOS) but also in regulating cell state, cell cycle, and mitochondrial homeostasis (Huang et al., 2020; Li et al., 2016). Xie et al. discovered that the increased expression of MRPL9 substantially enhances cell proliferation, metastasis, and disrupts the cell cycle by promoting the transition from G1 to S phase (Xie et al., 2022). The Mitochondrial Unfolded Protein Response (UPRmt) is a preserved stress response pathway triggered by protein misfolding and aggregation within mitochondria (Melber and Haynes, 2018). This pathway is linked to neurodegenerative diseases (Jovaisaite et al., 2014). MRPS6, functioning as a mitochondrial ribosome subunit, plays a vital role in the accurate translation of mitochondrial DNA. In individuals with DR, especially under high glucose conditions, it is crucial for the pancreas to tightly regulate mitochondrial metabolism. A recent study revealed that the overexpression of MRPS6 led to a decrease in the Mitochondrial Unfolded Protein Response (UPRmt), a reduction in high glucose-induced reactive oxygen species (ROS) levels and apoptosis, and an improvement in glucose-stimulated insulin secretion (GSIS) (Lin et al., 2023). Therefore, as a mitochondrial ribosomal protein that can be regulated by hyperglycemia, the effect of MRPS6 on improving insulin secretion and reducing ROS level is similar to that of metformin (Lu et al., 2016), which is currently a clinical hypoglycemic drug, suggesting that MRPS6 can be used as a new target for combination therapy. Ubiquinone oxidoreductase (NDU)-FAB1 is not just a crucial component of the mitochondrial fatty acid synthesis (mtFAS) pathway (Hiltunen et al., 2010), but also an auxiliary subunit of complex I (Vinothkumar et al., 2014). Furthermore, NDUFAB1 plays a role in iron-sulfur (FeS) biogenesis, with FeS centers crucial for the assembly of electron transport chain (ETC) complexes (Van Vranken et al., 2016). Zhang et al. showed that NDUFAB1 can boost mitochondrial metabolism and effectively combat metabolic stress (Zhang et al., 2019). Therefore, we hypothesize that the downregulation of NDUFAB1-associated genes correlates with oxidative stress in mitochondrial metabolic disorders among DR patients. The identification of hub genes also indicates the significant involvement of histones in DR. Nucleosomes are recognized as the fundamental units of chromatin, comprising octamers of histones H2A, H2B, H3, and H4, around which DNA is wrapped. Previous studies have revealed alterations in the expression of various genes crucial in retinal metabolic abnormalities among patients with DR (Biswas et al., 2019). Histones play a crucial role in governing gene expression, with post-translational modifications regulating transcription and genomic stability, such as histone acetylation, methylation, and phosphorylation (Bhaumik et al., 2007). Zhong et al. reported that exposure of endothelial cells, which are pathological targets linked to DR, to high glucose levels resulted in a decreased level of acetylated histone H3. This indicates the significant involvement of histone acetylation in the progression of DR (Zhong and Kowluru, 2010). In the study by Wang et al., it was discovered that diabetic rats exhibited several abnormal methylation markers in the H4 series within the retina in contrast to non-diabetic rats (Wang et al., 2017). The findings from these studies indicate that epigenetic modifications, especially histone post-translational modifications (PTM), are crucial in the progression of DR. Hence, PTM could serve as a delicate indicator for investigating the molecular mechanisms and diagnosis of DR. Furthermore, the observed dysregulation of histone acetylation in diabetic retinopathy (DR) patients underscores the therapeutic potential of histone deacetylase (HDAC) inhibitors. Mounting evidence from recent studies has demonstrated the clinical efficacy of HDAC inhibitors across various neurological disorders, including Parkinson’s disease and Alzheimer’s disease, among others (Zhang et al., 2024). Our findings suggest that HDAC-targeted interventions may offer both prophylactic and therapeutic benefits for DR, potentially expanding the clinical applications of this drug class.

The findings from CSEA indicated that genes associated with DR were uniquely expressed in the cerebral cortex. The brain is intricately linked to distinct sensory organs, and visual information captured by our eyes is transmitted to specialized neurons in the visual cortex of the brain (Masland, 2012). DR is a neurodegenerative disease affecting the microvasculature, leading to visual impairment. Clinical evidence indicates a correlation between central nervous system (CNS) lesions, retinopathy, and brain tissue damage in patients with DR (Wang et al., 2017). Concurrently, patients with DR often exhibit cognitive impairment and an elevated risk of dementia and Alzheimer’s disease due to central nervous system abnormalities (Huang et al., 2019). At the same time, the downregulated genes within the amygdala, cerebellum, cortex, hippocampus, striatum, and thalamus were observed during both adolescent and adult developmental stages, thereby substantiating the link between DR and the central nervous system of the brain. The amygdala and hippocampus play essential roles in the formation of spatial, episodic, declarative, and emotional memories, as well as in motivational processes (McDonald and Mott, 2017). The cerebellum plays a critical role in maintaining body balance, coordinating motor movements, and controlling eye movements (Herzfeld et al., 2015). The striatum plays a significant role in the pathway of memory encoding (Pennartz et al., 2011). The thalamus has long been recognized as a crucial intermediary in transmitting information from the periphery to the cortex, facilitating the relay of visual, auditory, and somatosensory signals (Kerschensteiner and Guido, 2017). The functions of these brain regions support the hypothesis that the visual and cognitive impairments frequently observed in patients with DR may be associated with central neurodegeneration. The enrichment results imply that the differential genes linked to these functions could offer a valuable genetic basis for investigating VMHC alterations in individuals with DR. The shared genetic loci between cerebral dysfunction and retinal neurodegeneration substantiate DR as a systemic metabolic disorder. Clinically, therapeutic strategies for DR should incorporate comprehensive evaluation of potential central nervous system complications to optimize diagnostic precision and treatment efficacy.

Several limitations should be taken into account when interpreting our findings. First, the AHBA gene expression data and VMHC measures used for transcriptomic-neuroimaging association analyses were derived from different cohorts, limiting our study to examine individual-specific relationships. Future research should develop integrated models incorporating patient-specific transcriptomic profiles to validate these findings. Secondly, VMHC does not capture all facets of brain functional homotopy, including the synchronization of spontaneous activity during resting states and the co-activation among task-induced homotopy regions; VMHC characterizes the former, not the latter. The genetic underpinnings of other metrics that reflect brain functional homotopy also warrant further investigation. Thirdly, head movements during rs-fMRI scans could compromise the quality and interpretability of the collected data. Although the study conducted quality checks and implemented head movement correction, it is essential to recognize that the residual effects of head motion may not have been entirely eradicated. Fourthly, in the present study, only left-hemisphere cortical tissue samples were analyzed. Given that VMHC reflects functional synchronization between homologous bilateral brain regions, its alterations may be regulated by the coordinated expression of genes in both hemispheres. Thus, investigations focusing exclusively on unilateral hemispheric gene expression provide an incomplete perspective on disease mechanisms. Future studies should incorporate bilateral transcriptome data to better elucidate the genetic underpinnings of VMHC changes. Ultimately, the relatively limited sample size and absence of certain clinical assessments could impede the statistical power to detect alterations in brain function and elucidate the relationships between genes, brain activity, and behavior. A larger sample size is crucial for increasing the statistical power of our analyses. It would enable us to detect more subtle changes in brain function and establish more reliable relationships between genes, brain activity, and behavior. Additionally, comprehensive clinical assessments in a large - scale study would provide valuable context for understanding these relationships, facilitating the translation of our findings into clinical practice. Consequently, future research endeavors should encompass larger cohorts of patients with DR, along with comprehensive clinical data, to authenticate our results.

## 5 Conclusion

As the pioneering study to integrate a thorough examination of brain imaging and gene expression data in exploring the genetic underpinnings of VMHC changes in individuals with DR, we identified distinctive modifications in VMHC within various brain regions, such as the bilateral LING, PoCG, and PreCG. Through the application of transcription-neuroimaging spatial associations to rs-fMRI data from extensive discovery and validation samples, our findings elucidate the genetic control of brain function homomorphism. The findings demonstrate that impaired interhemispheric connectivity in DR involves complex interactions among genes regulating neurovascular, metabolic, and neurodegenerative pathways. Our findings not only provide a unique perspective on the genetic mechanism of VMHC alteration in DR Patients, but also provide new research directions for disease diagnosis, treatment and intervention from the perspective of molecular mechanisms. In summary, this study establishes a connection between fMRI phenotypes and gene expression, unveiling the neurobiological genetic signature that underlies brain abnormalities in DR individuals.

## Data Availability

The raw data supporting the conclusions of this article will be made available by the authors, without undue reservation.
