# Supplementary material for: Genetic mechanisms of hemispheric functional connectivity in diabetic retinopathy: a joint neuroimaging and transcriptomic study
Source: Front Cell Dev Biol. 2025 May 6;13:1590627. doi: 10.3389/fcell.2025.1590627 (PMC12096415; doi:10.3389/fcell.2025.1590627)
Supplement: Supplementary file 1 [file DataSheet3.docx]

| Figure 4A/4C | | | |
| --- | --- | --- | --- |
| Term | Count | PValue | group |
| positive regulation of transcription elongation by RNA polymerase II | 18 | 2.07E-07 | BP |
| mitochondrial translation | 23 | 1.01E-05 | BP |
| microtubule-based movement | 20 | 1.72E-05 | BP |
| fatty acid metabolic process | 22 | 2.05E-05 | BP |
| lipid phosphorylation | 7 | 9.36E-05 | BP |
| ubiquitin-dependent protein catabolic process via the C-end degron rule pathway | 7 | 1.74E-04 | BP |
| response to methylmercury | 5 | 2.05E-04 | BP |
| cytosol | 571 | 8.10E-12 | CC |
| cytoplasm | 577 | 2.39E-11 | CC |
| glutamatergic synapse | 77 | 3.93E-11 | CC |
| mitochondrion | 182 | 2.76E-09 | CC |
| nucleoplasm | 393 | 3.82E-06 | CC |
| core mediator complex | 12 | 4.75E-06 | CC |
| mitochondrial large ribosomal subunit | 17 | 6.07E-06 | CC |
| protein binding | 1216 | 2.12E-12 | MF |
| ATP hydrolysis activity | 72 | 7.32E-08 | MF |
| ATP binding | 191 | 8.31E-08 | MF |
| hydrolase activity | 40 | 5.38E-05 | MF |
| microtubule binding | 44 | 9.29E-05 | MF |
| microtubule motor activity | 16 | 9.55E-05 | MF |
| ATP-dependent diacylglycerol kinase activity | 7 | 1.22E-04 | MF |

| Figure 4E | | | | |
| --- | --- | --- | --- | --- |
| Term | Fold Enrichment | PValue | Count | group |
| Metabolic pathways | 1.533592497 | 3.03E-12 | 218 | KEGG |
| Lysosome | 2.495765104 | 5.48E-06 | 30 | KEGG |
| Pertussis | 2.956521739 | 1.44E-05 | 21 | KEGG |
| Phosphatidylinositol signaling system | 2.689314235 | 1.64E-05 | 24 | KEGG |
| Motor proteins | 2.062490147 | 3.48E-05 | 37 | KEGG |
| Epithelial cell signaling in Helicobacter pylori infection | 2.784008398 | 1.57E-04 | 18 | KEGG |
| Pyruvate metabolism | 3.271045328 | 2.00E-04 | 14 | KEGG |
| Pathways in cancer | 1.504014543 | 3.36E-04 | 73 | KEGG |
| Pathways of neurodegeneration - multiple diseases | 1.500559392 | 7.33E-04 | 66 | KEGG |
| Circadian entrainment | 2.26419927 | 0.001015855 | 20 | KEGG |
| Legionellosis | 2.745341615 | 0.001246362 | 14 | KEGG |
| Glycosaminoglycan biosynthesis - chondroitin sulfate / dermatan sulfate | 4.183377699 | 0.001867694 | 8 | KEGG |
| Salmonella infection | 1.662517631 | 0.002137566 | 38 | KEGG |
| Aldosterone synthesis and secretion | 2.129040436 | 0.002887552 | 19 | KEGG |
| Kaposi sarcoma-associated herpesvirus infection | 1.736848777 | 0.003076858 | 31 | KEGG |

| Figure 4B/4D | | | |
| --- | --- | --- | --- |
| Term | Count | PValue | group |
| negative regulation of transcription by RNA polymerase II | 115 | 3.15E-05 | BP |
| nervous system development | 59 | 3.55E-05 | BP |
| cell-cell adhesion | 36 | 6.38E-05 | BP |
| protein processing | 19 | 1.95E-04 | BP |
| neurotransmitter receptor localization to postsynaptic specialization membrane | 8 | 2.22E-04 | BP |
| cytoplasmic microtubule organization | 14 | 2.57E-04 | BP |
| mitochondrial respiratory chain complex I assembly | 16 | 2.64E-04 | BP |
| cytosol | 595 | 1.24E-14 | CC |
| cytoplasm | 588 | 7.73E-12 | CC |
| mitochondrion | 185 | 1.91E-09 | CC |
| nucleoplasm | 418 | 7.28E-09 | CC |
| membrane | 493 | 6.25E-07 | CC |
| mitochondrial inner membrane | 70 | 8.91E-06 | CC |
| beta-catenin destruction complex | 8 | 8.04E-05 | CC |
| protein binding | 1254 | 2.69E-20 | MF |
| voltage-gated potassium channel activity | 15 | 2.41E-05 | MF |
| actin filament binding | 40 | 5.45E-05 | MF |
| RNA polymerase II-specific DNA-binding transcription factor binding | 31 | 4.42E-04 | MF |
| integrin binding | 28 | 5.88E-04 | MF |
| protein homodimerization activity | 91 | 5.94E-04 | MF |
| MAP kinase tyrosine/serine/threonine phosphatase activity | 7 | 6.35E-04 | MF |

| Figure 4F | | | | |
| --- | --- | --- | --- | --- |
| Term | Fold Enrichment | PValue | Count | group |
| Metabolic pathways | 1.355256602 | 1.59E-06 | 196 | KEGG |
| Various types of N-glycan biosynthesis | 3.854875283 | 1.36E-05 | 15 | KEGG |
| N-Glycan biosynthesis | 3.054806828 | 2.35E-04 | 15 | KEGG |
| Regulation of actin cytoskeleton | 1.783298827 | 5.74E-04 | 38 | KEGG |
| Thermogenesis | 1.607565012 | 0.005607792 | 35 | KEGG |
| Autophagy - other | 3.035714286 | 0.007320737 | 9 | KEGG |
| Notch signaling pathway | 2.263184844 | 0.010328028 | 13 | KEGG |
| Lysine degradation | 2.227261275 | 0.011717518 | 13 | KEGG |
| Hedgehog signaling pathway | 2.31292517 | 0.012351025 | 12 | KEGG |
| Fatty acid metabolism | 2.272347536 | 0.014065599 | 12 | KEGG |
| Pathways of neurodegeneration - multiple diseases | 1.340826186 | 0.015480182 | 60 | KEGG |
| Fc gamma R-mediated phagocytosis | 1.872367995 | 0.017341106 | 17 | KEGG |
| Cysteine and methionine metabolism | 2.283272283 | 0.019210372 | 11 | KEGG |
| Other glycan degradation | 3.597883598 | 0.020698485 | 6 | KEGG |
| Fatty acid biosynthesis | 3.597883598 | 0.020698485 | 6 | KEGG |
