# Supplementary material for: Genetic mechanisms of hemispheric functional connectivity in diabetic retinopathy: a joint neuroimaging and transcriptomic study
Source: Front Cell Dev Biol. 2025 May 6;13:1590627. doi: 10.3389/fcell.2025.1590627 (PMC12096415; doi:10.3389/fcell.2025.1590627)
Supplement: Supplementary file 2 [file DataSheet2.docx]

| Figure 8A | | | | |
| --- | --- | --- | --- | --- |
| Brain Cell Types and P-Values | 0.05 | 0.01 | 0.001 | 0.0001 |
| RetR.Rods | 0.994 | 0.942 | 0.787 | 0.944 |
| Hyp | 0.538 | 0.755 | 0.746 | 0.394 |
| Hyp.Hcrt | 0.55 | 0.637 | 0.323 | 0.461 |
| BF | 0.876 | 0.299 | 0.625 | 1 |
| BF.Chat | 0.968 | 0.723 | 0.935 | 0.909 |
| BS | 0.833 | 0.582 | 1 | 1 |
| BS.Chat | 0.753 | 0.655 | 0.861 | 0.928 |
| BS.Slc6a4 | 0.813 | 0.938 | 0.973 | 0.927 |
| Cb | 0.922 | 0.475 | 0.922 | 1 |
| Cb.Septin4 | 0.308 | 0.703 | 0.688 | 0.982 |
| Cb.Pcp2 | 0.897 | 0.922 | 0.942 | 0.807 |
| Cb.Neurod1 | 0.769 | 0.237 | 0.244 | 1 |
| Cb.Lypd6 | 0.485 | 0.513 | 0.598 | 0.54 |
| Cb.Grp | 0.889 | 0.884 | 0.582 | 0.679 |
| Cb.Grm2 | 0.754 | 0.618 | 0.146 | 0.536 |
| Cb.Fthfd | 0.585 | 0.907 | 0.855 | 0.966 |
| Cb.Cnp | 0.082 | 0.234 | 0.444 | 0.387 |
| Cpu | 0.977 | 0.879 | 0.582 | 0.279 |
| Cpu.D2 | 0.739 | 0.965 | 0.999 | 0.969 |
| Cpu.D1 | 0.992 | 0.988 | 0.933 | 1 |
| Cpu.Chat | 0.758 | 0.748 | 0.857 | 0.857 |
| Ctx | 0.656 | 0.982 | 1 | 1 |
| Ctx.Pdgfrajd340 | 0.914 | 0.97 | 0.964 | 1 |
| Ctx.Etv1_ts88 | 0.645 | 0.991 | 0.999 | 1 |
| Ctx.Pnoc | 0.091 | 0.224 | 0.333 | 0.282 |
| Ctx.Ntsr | 0.801 | 0.032 | 0.394 | 0.48 |
| Ctx.Glt25d2 | 0.375 | 0.702 | 1 | 1 |
| Ctx.Fthfd | 0.565 | 0.543 | 0.526 | 0.628 |
| Ctx.Cort | 0.15 | 0.356 | 0.843 | 1 |
| Ctx.Cnp | 0.082 | 0.207 | 0.246 | 0.167 |
| Epi | 0.856 | 0.677 | 0.394 | 0.934 |
| Epi.Chat | 0.715 | 0.602 | 0.4 | 0.436 |
| Spc | 0.035 | 0.892 | 0.699 | 0.8 |
| Spc.Chat | 0.34 | 0.212 | 0.778 | 0.517 |
| RetC.Cones | 0.842 | 0.918 | 0.901 | 0.875 |
|  |  |  |  |  |
| Figure 8B | | | | |
| Brain Regions and Development and P-Values | 0.05 | 0.01 | 0.001 | 0.0001 |
| Amygdala.Adolescence | 7.13E-05 | 0.445 | 0.721 | 1 |
| Cerebellum.Adolescence | 0.018 | 0.052 | 0.611 | 0.481 |
| Cortex.Adolescence | 1.00E-05 | 0.003 | 0.587 | 1 |
| Hippocampus.Adolescence | 0.009 | 0.095 | 1 | 1 |
| Striatum.Adolescence | 0.016 | 0.019 | 0.307 | 0.072 |
| Thalamus.Adolescence | 0.011 | 0.388 | 0.26 | 1 |
| Amygdala.Early.Childhood | 0.147 | 1 | 1 | 1 |
| Cerebellum.Early.Childhood | 0.094 | 0.416 | 0.873 | 1 |
| Cortex.Early.Childhood | 0.011 | 0.671 | 1 | 1 |
| Hippocampus.Early.Childhood | 0.029 | 0.946 | 1 | 1 |
| Striatum.Early.Childhood | 0.17 | 0.669 | 0.059 | 1 |
| Thalamus.Early.Childhood | 0.015 | 0.526 | 1 | 1 |
| Amygdala.Early.Fetal | 0.6 | 0.943 | 0.626 | 1 |
| Cerebellum.Early.Fetal | 0.686 | 0.811 | 0.853 | 0.936 |
| Cortex.Early.Fetal | 0.964 | 0.971 | 0.83 | 1 |
| Hippocampus.Early.Fetal | 0.623 | 0.999 | 0.936 | 1 |
| Striatum.Early.Fetal | 0.64 | 0.904 | 0.989 | 0.812 |
| Thalamus.Early.Fetal | 0.359 | 0.562 | 0.84 | 1 |
| Amygdala.Early.Mid.Fetal | 0.943 | 0.716 | 1 | 1 |
| Cerebellum.Early.Mid.Fetal | 0.721 | 0.908 | 0.998 | 1 |
| Cortex.Early.Mid.Fetal | 0.443 | 0.848 | 0.692 | 1 |
| Hippocampus.Early.Mid.Fetal | 0.969 | 0.581 | 0.497 | 0.325 |
| Striatum.Early.Mid.Fetal | 0.728 | 0.598 | 0.26 | 1 |
| Thalamus.Early.Mid.Fetal | 0.679 | 0.668 | 0.845 | 1 |
| Amygdala.Late.Fetal | 0.893 | 0.449 | 0.544 | 1 |
| Cerebellum.Late.Fetal | 0.432 | 0.983 | 1 | 1 |
| Cortex.Late.Fetal | 0.121 | 0.531 | 1 | 1 |
| Hippocampus.Late.Fetal | 0.996 | 0.81 | 0.388 | 1 |
| Striatum.Late.Fetal | 0.999 | 0.982 | 0.721 | 1 |
| Thalamus.Late.Fetal | 0.666 | 0.833 | 0.715 | 0.771 |
| Amygdala.Late.Infancy | 0.16 | 0.247 | 0.544 | 1 |
| Cerebellum.Late.Infancy | 0.052 | 0.646 | 0.876 | 0.84 |
| Cortex.Late.Infancy | 0.028 | 0.914 | 0.255 | 1 |
| Hippocampus.Late.Infancy | 0.09 | 0.994 | 0.83 | 1 |
| Striatum.Late.Infancy | 0.115 | 0.697 | 1 | 1 |
| Thalamus.Late.Infancy | 0.305 | 0.254 | 1 | 1 |
| Amygdala.Late.Mid.Fetal | 0.837 | 0.963 | 1 | 1 |
| Cerebellum.Late.Mid.Fetal | 0.998 | 0.958 | 1 | 1 |
| Cortex.Late.Mid.Fetal | 0.011 | 0.021 | 1 | 1 |
| Hippocampus.Late.Mid.Fetal | 0.989 | 0.984 | 0.733 | 1 |
| Striatum.Late.Mid.Fetal | 0.413 | 0.681 | 0.885 | 1 |
| Thalamus.Late.Mid.Fetal | 0.984 | 0.904 | 0.824 | 0.685 |
| Amygdala.Middle.Late.Childhood | 0.074 | 0.93 | 1 | 1 |
| Cerebellum.Middle.Late.Childhood | 0.014 | 0.156 | 0.648 | 1 |
| Cortex.Middle.Late.Childhood | 0.136 | 0.096 | 0.512 | 1 |
| Hippocampus.Middle.Late.Childhood | 0.113 | 0.526 | 0.692 | 1 |
| Striatum.Middle.Late.Childhood | 0.22 | 0.537 | 0.733 | 0.626 |
| Thalamus.Middle.Late.Childhood | 0.015 | 0.366 | 0.968 | 0.792 |
| Amygdala.Neotal.Early.Infancy | 0.696 | 0.733 | 0.178 | 1 |
| Cerebellum.Neotal.Early.Infancy | 0.123 | 0.434 | 0.781 | 0.828 |
| Cortex.Neotal.Early.Infancy | 0.379 | 0.727 | 0.416 | 1 |
| Hippocampus.Neotal.Early.Infancy | 0.608 | 0.518 | 1 | 1 |
| Striatum.Neotal.Early.Infancy | 0.861 | 0.722 | 1 | 1 |
| Thalamus.Neotal.Early.Infancy | 0.074 | 0.041 | 0.329 | 0.325 |
| Amygdala.Young.Adulthood | 0.019 | 0.208 | 1 | 1 |
| Cerebellum.Young.Adulthood | 1.30E-04 | 0.23 | 0.172 | 0.769 |
| Cortex.Young.Adulthood | 5.09E-07 | 0.057 | 0.095 | 0.275 |
| Hippocampus.Young.Adulthood | 0.019 | 0.995 | 0.896 | 0.587 |
| Striatum.Young.Adulthood | 1.95E-04 | 0.264 | 1 | 1 |
| Thalamus.Young.Adulthood | 0.002 | 0.094 | 0.449 | 0.255 |
|  |  |  |  |  |
| Figure 8C | | | | |
| Brain Regions and Development and P-Values | 0.05 | 0.01 | 0.001 | 0.0001 |
| Amygdala.Young.Adulthood | 0.24 | 0.873 | 1 | 1 |
| Cerebellum.Young.Adulthood | 0.003 | 0.151 | 0.871 | 0.74 |
| Cortex.Young.Adulthood | 0.002 | 0.06 | 0.542 | 1 |
| Hippocampus.Young.Adulthood | 0.88 | 0.743 | 0.992 | 1 |
| Striatum.Young.Adulthood | 0.181 | 0.729 | 0.775 | 0.845 |
| Thalamus.Young.Adulthood | 0.048 | 0.003 | 0.804 | 1 |
