# Supplementary material for: Genetic mechanisms of hemispheric functional connectivity in diabetic retinopathy: a joint neuroimaging and transcriptomic study
Source: Front Cell Dev Biol. 2025 May 6;13:1590627. doi: 10.3389/fcell.2025.1590627 (PMC12096415; doi:10.3389/fcell.2025.1590627)
Supplement: Supplementary file 3 [file DataSheet1.docx]

| Figure 7A | | | | |
| --- | --- | --- | --- | --- |
| Brain Cell Types and P-Values | 0.05 | 0.01 | 0.001 | 0.0001 |
| RetR.Rods | 0.801 | 0.976 | 0.771 | 0.74 |
| Hyp | 0.999 | 0.951 | 1 | 1 |
| Hyp.Hcrt | 0.81 | 0.725 | 0.46 | 0.647 |
| BF | 0.475 | 0.946 | 1 | 1 |
| BF.Chat | 0.895 | 0.905 | 0.609 | 0.669 |
| BS | 0.811 | 0.985 | 1 | 1 |
| BS.Chat | 0.578 | 0.63 | 0.851 | 0.589 |
| BS.Slc6a4 | 0.968 | 0.998 | 0.971 | 0.924 |
| Cb | 0.871 | 0.679 | 0.362 | 0.386 |
| Cb.Septin4 | 0.976 | 0.996 | 0.988 | 0.98 |
| Cb.Pcp2 | 0.835 | 0.859 | 0.875 | 0.949 |
| Cb.Neurod1 | 0.79 | 0.216 | 0.8 | 0.558 |
| Cb.Lypd6 | 0.907 | 0.995 | 0.969 | 0.981 |
| Cb.Grp | 0.948 | 0.978 | 0.772 | 0.905 |
| Cb.Grm2 | 0.971 | 0.999 | 0.883 | 0.838 |
| Cb.Fthfd | 0.993 | 1 | 0.924 | 0.622 |
| Cb.Cnp | 0.969 | 0.6 | 0.321 | 0.255 |
| Cpu | 0.071 | 0.261 | 1 | 1 |
| Cpu.D2 | 0.088 | 0.334 | 0.338 | 0.411 |
| Cpu.D1 | 0.007 | 0.394 | 0.19 | 0.446 |
| Cpu.Chat | 0.934 | 0.957 | 1 | 1 |
| Ctx | 0.536 | 0.526 | 1 | 1 |
| Ctx.Pdgfrajd340 | 0.681 | 0.634 | 0.49 | 0.092 |
| Ctx.Etv1_ts88 | 0.168 | 0.279 | 0.494 | 0.32 |
| Ctx.Pnoc | 1 | 0.992 | 0.902 | 1 |
| Ctx.Ntsr | 0.002 | 0.21 | 0.036 | 0.474 |
| Ctx.Glt25d2 | 0.089 | 0.687 | 0.751 | 1 |
| Ctx.Fthfd | 0.994 | 0.997 | 0.995 | 0.958 |
| Ctx.Cort | 0.966 | 0.684 | 1 | 1 |
| Ctx.Cnp | 0.912 | 0.586 | 0.225 | 0.38 |
| Epi | 0.834 | 0.927 | 0.995 | 0.931 |
| Epi.Chat | 0.788 | 0.983 | 0.995 | 0.978 |
| Spc | 0.82 | 0.879 | 0.982 | 1 |
| Spc.Chat | 0.431 | 0.363 | 0.321 | 0.722 |
| RetC.Cones | 0.724 | 0.924 | 0.956 | 0.982 |
|  |  |  |  |  |
| Figure 7B | | | | |
| Amygdala.Adolescence | 0.836 | 0.795 | 0.348 | 1 |
| Cerebellum.Adolescence | 0.328 | 0.618 | 0.27 | 0.21 |
| Cortex.Adolescence | 0.507 | 0.957 | 0.588 | 1 |
| Hippocampus.Adolescence | 0.559 | 0.995 | 1 | 1 |
| Striatum.Adolescence | 0.094 | 0.334 | 0.544 | 0.389 |
| Thalamus.Adolescence | 0.136 | 0.714 | 0.846 | 0.256 |
| Amygdala.Early.Childhood | 0.852 | 1 | 1 | 1 |
| Cerebellum.Early.Childhood | 0.527 | 0.973 | 0.264 | 0.169 |
| Cortex.Early.Childhood | 0.131 | 0.672 | 1 | 1 |
| Hippocampus.Early.Childhood | 0.31 | 0.986 | 1 | 1 |
| Striatum.Early.Childhood | 0.459 | 0.522 | 1 | 1 |
| Thalamus.Early.Childhood | 0.752 | 0.959 | 1 | 1 |
| Amygdala.Early.Fetal | 0.133 | 0.46 | 0.627 | 1 |
| Cerebellum.Early.Fetal | 0.65 | 0.739 | 0.998 | 0.937 |
| Cortex.Early.Fetal | 0.312 | 0.261 | 1 | 1 |
| Hippocampus.Early.Fetal | 0.223 | 0.969 | 0.937 | 0.627 |
| Striatum.Early.Fetal | 0.005 | 0.036 | 0.679 | 1 |
| Thalamus.Early.Fetal | 0.831 | 0.746 | 0.842 | 1 |
| Amygdala.Early.Mid.Fetal | 0.015 | 0.577 | 1 | 1 |
| Cerebellum.Early.Mid.Fetal | 0.837 | 0.981 | 0.964 | 0.183 |
| Cortex.Early.Mid.Fetal | 0.182 | 0.431 | 0.693 | 1 |
| Hippocampus.Early.Mid.Fetal | 0.595 | 0.781 | 0.498 | 1 |
| Striatum.Early.Mid.Fetal | 0.445 | 0.601 | 0.544 | 1 |
| Thalamus.Early.Mid.Fetal | 0.055 | 1 | 1 | 1 |
| Amygdala.Late.Fetal | 0.24 | 0.452 | 0.545 | 1 |
| Cerebellum.Late.Fetal | 0.927 | 0.413 | 0.599 | 1 |
| Cortex.Late.Fetal | 0.354 | 0.243 | 0.446 | 1 |
| Hippocampus.Late.Fetal | 0.671 | 0.988 | 1 | 1 |
| Striatum.Late.Fetal | 0.41 | 0.681 | 0.722 | 1 |
| Thalamus.Late.Fetal | 0.046 | 0.301 | 0.869 | 0.772 |
| Amygdala.Late.Infancy | 0.037 | 0.959 | 1 | 1 |
| Cerebellum.Late.Infancy | 0.322 | 0.87 | 0.962 | 0.842 |
| Cortex.Late.Infancy | 0.459 | 0.915 | 1 | 1 |
| Hippocampus.Late.Infancy | 0.445 | 0.654 | 0.83 | 1 |
| Striatum.Late.Infancy | 0.149 | 0.3 | 0.514 | 0.389 |
| Thalamus.Late.Infancy | 0.026 | 0.975 | 1 | 1 |
| Amygdala.Late.Mid.Fetal | 0.759 | 0.638 | 1 | 1 |
| Cerebellum.Late.Mid.Fetal | 0.912 | 0.641 | 0.995 | 1 |
| Cortex.Late.Mid.Fetal | 0.056 | 0.249 | 1 | 1 |
| Hippocampus.Late.Mid.Fetal | 0.8 | 0.94 | 0.735 | 1 |
| Striatum.Late.Mid.Fetal | 0.64 | 0.788 | 0.886 | 1 |
| Thalamus.Late.Mid.Fetal | 0.78 | 0.987 | 0.998 | 0.992 |
| Amygdala.Middle.Late.Childhood | 0.972 | 0.735 | 1 | 1 |
| Cerebellum.Middle.Late.Childhood | 0.507 | 0.929 | 0.853 | 0.662 |
| Cortex.Middle.Late.Childhood | 0.641 | 0.979 | 1 | 1 |
| Hippocampus.Middle.Late.Childhood | 0.981 | 1 | 1 | 1 |
| Striatum.Middle.Late.Childhood | 0.842 | 0.986 | 1 | 1 |
| Thalamus.Middle.Late.Childhood | 0.838 | 0.975 | 0.853 | 1 |
| Amygdala.Neotal.Early.Infancy | 0.626 | 1 | 1 | 1 |
| Cerebellum.Neotal.Early.Infancy | 0.32 | 0.824 | 0.907 | 0.961 |
| Cortex.Neotal.Early.Infancy | 0.386 | 0.947 | 1 | 1 |
| Hippocampus.Neotal.Early.Infancy | 0.389 | 0.77 | 1 | 1 |
| Striatum.Neotal.Early.Infancy | 0.117 | 0.906 | 0.83 | 1 |
| Thalamus.Neotal.Early.Infancy | 0.027 | 0.979 | 1 | 1 |
| Amygdala.Young.Adulthood | 0.602 | 0.63 | 1 | 1 |
| Cerebellum.Young.Adulthood | 0.574 | 0.916 | 0.957 | 0.943 |
| Cortex.Young.Adulthood | 0.438 | 0.958 | 0.544 | 0.276 |
| Hippocampus.Young.Adulthood | 0.946 | 0.978 | 1 | 1 |
| Striatum.Young.Adulthood | 0.602 | 0.268 | 0.027 | 1 |
| Thalamus.Young.Adulthood | 0.59 | 0.675 | 0.793 | 0.256 |
|  |  |  |  |  |
| Figure 7C | | | | |
| Brain Regions and Development and P-Values | 0.05 | 0.01 | 0.001 | 0.0001 |
| Amygdala.Young.Adulthood | 0.637 | 0.314 | 1 | 1 |
| Cerebellum.Young.Adulthood | 0.66 | 0.864 | 0.475 | 0.742 |
| Cortex.Young.Adulthood | 0.246 | 0.212 | 0.544 | 0.446 |
| Hippocampus.Young.Adulthood | 0.884 | 0.999 | 0.952 | 0.793 |
| Striatum.Young.Adulthood | 0.188 | 0.65 | 0.892 | 0.544 |
| Thalamus.Young.Adulthood | 0.666 | 0.913 | 0.919 | 0.896 |
